# Antiplatelet Activity, P2Y_1_ and P2Y_12_ Inhibition, and Metabolism in Plasma of Stereoisomers of Diadenosine 5′,5′″-P^1^,P^4^-dithio-P^2^,P^3^-chloromethylenetetraphosphate

**DOI:** 10.1371/journal.pone.0094780

**Published:** 2014-04-10

**Authors:** Hung Chang, Ivan B. Yanachkov, Edward J. Dix, Milka Yanachkova, YouFu Li, Marc R. Barnard, George E. Wright, Alan D. Michelson, Andrew L. Frelinger

**Affiliations:** 1 Center for Platelet Function Studies, Department of Pediatrics, University of Massachusetts Medical School, Worcester, Massachusetts, United States of America; 2 Hematology Division, Chang Gung Memorial Hospital, Chang Gung University, Taipei, Taiwan; 3 GLSynthesis Inc., Worcester, Massachusetts, United States of America; 4 Center for Platelet Research Studies, Division of Hematology/Oncology, Boston Children's Hospital, Dana-Farber Cancer Institute, Harvard Medical School, Boston, Massachusetts, United States of America; Royal College of Surgeons, Ireland

## Abstract

**Background:**

Diadenosine tetraphosphate (Ap_4_A), a constituent of platelet dense granules, and its P^1^,P^4^-dithio and/or P^2^,P^3^-chloromethylene analogs, inhibit adenosine diphosphate (ADP)-induced platelet aggregation. We recently reported that these compounds antagonize both platelet ADP receptors, P2Y_1_ and P2Y_12_. The most active of those analogs, diadenosine 5′,5″″-P^1^,P^4^-dithio-P^2^,P^3^-chloromethylenetetraphosphate, (compound **1**), exists as a mixture of 4 stereoisomers.

**Objective:**

To separate the stereoisomers of compound **1** and determine their effects on platelet aggregation, platelet P2Y_1_ and P2Y_12_ receptor antagonism, and their metabolism in human plasma.

**Methods:**

We separated the 4 diastereomers of compound **1** by preparative reversed-phase chromatography, and studied their effect on ADP-induced platelet aggregation, P2Y_1_-mediated changes in cytosolic Ca^2+^, P2Y_12_-mediated changes in VASP phosphorylation, and metabolism in human plasma.

**Results:**

The inhibition of ADP-induced human platelet aggregation and human platelet P2Y_12_ receptor, and stability in human plasma strongly depended on the stereo-configuration of the chiral P^1^- and P^4^-phosphorothioate groups, the S_P_S_P_ diastereomer being the most potent inhibitor and completely resistant to degradation in plasma, and the R_P_R_P_ diastereomer being the least potent inhibitor and with the lowest plasma stability. The inhibitory activity of S_P_R_P_ diastereomers depended on the configuration of the pseudo-asymmetric carbon of the P^2^,P^3^-chloromethylene group, one of the configurations being significantly more active than the other. Their plasma stability did not differ significantly, being intermediate to that of the S_P_S_P_ and the R_P_R_P_ diastereomers.

**Conclusions:**

The presently-described stereoisomers have utility for structural, mechanistic, and drug development studies of dual antagonists of platelet P2Y_1_ and P2Y_12_ receptors.

## Introduction

Platelets express two G-protein-coupled P2Y (nucleotide activated) receptors, P2Y_1_ and P2Y_12_ [1]. Both receptors are activated by adenosine 5′-diphosphate (ADP) and play essential and mutually dependent roles in the process of platelet activation and aggregation [1]. G_q_ coupled P2Y_1_ activates the beta-isoform of phospholipase C (PLCβ) and causes inositol 1,4,5-trisphosphate (IP3)-mediated increase in intracellular calcium levels, mainly by calcium release from intracellular stores. P2Y_1_ activation initiates ADP-induced platelet aggregation and results in platelet shape change [1]. However, without P2Y_12_ activation, the result is a small and reversible platelet aggregation. G_i_-coupled P2Y_12_ inhibits adenylyl cyclase, thereby stimulating phosphatidylinositol-3 kinase (PI-3K) activity. Reduced cAMP levels further reduce cAMP dependent protein kinase A phosphorylation of vasodilator-stimulated phosphoprotein (VASP), a modulator of platelet cytoskeletal proteins [2]. Functionally this results in potentiation of platelet secretion, and amplification and stabilization of the aggregation response. There is a complex interplay between P2Y_1_ and P2Y_12_, and co-activation of both receptors, or the signaling pathways they trigger is necessary for full platelet aggregation to take place [3].

**Figure 1:**
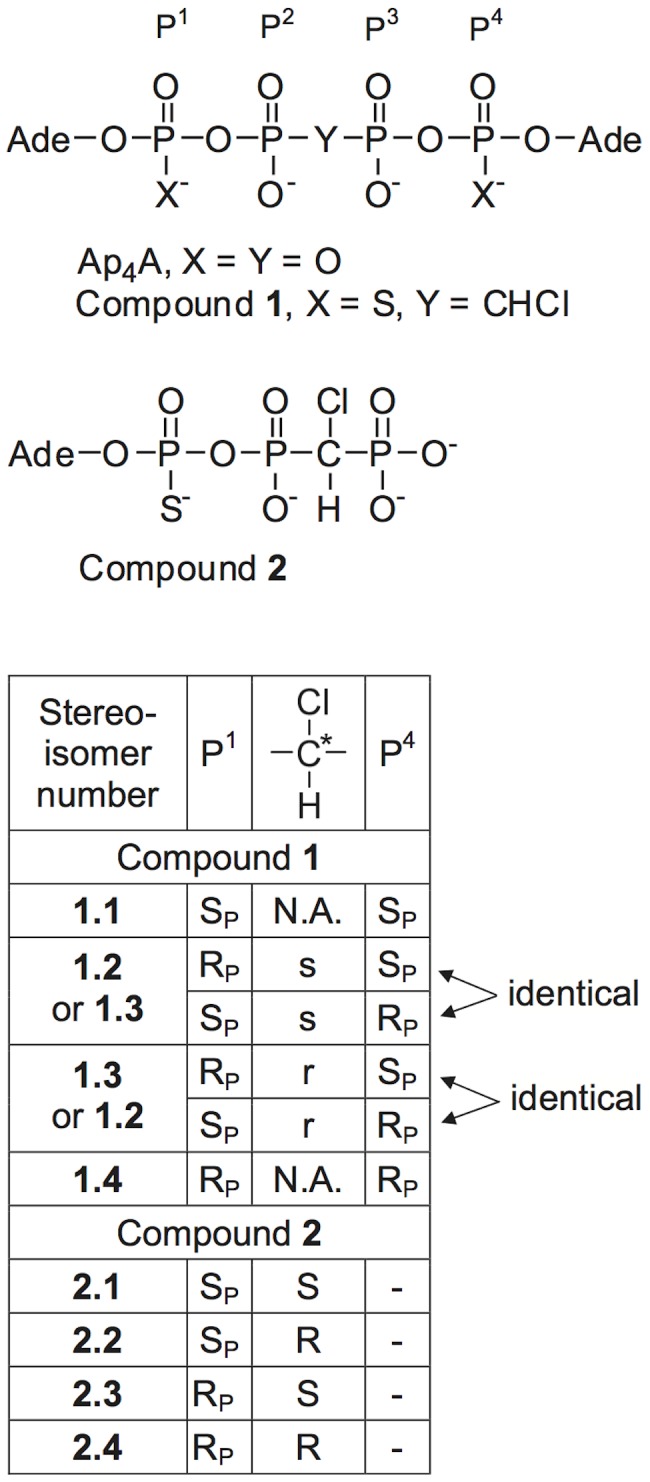
Chemical structure and stereo-configuration of the stereoisomers of diadenosine 5′,5″″-P^1^,P^4^-dithio-P^2^,P^3^-chloromethylenetetraphosphate (compound 1), and of adenosine 5′-(P^1^-thio-P^2^,P^3^-chloromethylenetriphosphate), (compound 2). R_P_ and S_P_ designate the absolute configuration of chiral P^1^- and P^4^-phosphorothioates; r and s, the absolute configuration of the pseudo-asymmetric carbon of the P^2^,P^3^-chloromethylene group in compound **1**; R and S, the absolute configuration of the chloromethylene group in compound **2**. Ade, 5′-adenosyl; N.A., Not Asymmetric.

Diadenosine 5′,5′″-tetraphosphate (Ap_4_A, Figure 1) is the most important member of the group of dinucleoside polyphosphates. It is found in a variety of cells, is secreted extracellularly, and is involved in the regulation of variety of intra- and extracellular physiological functions [4]. In platelets Ap_4_A is stored in dense granules and is therefore released along with ADP and ATP upon platelet activation [5]. Early studies of diadenosine polyphosphates found that diadenosine triphosphate induces platelet aggregation and that Ap_4_A antagonizes this effect [6]. It is now well known that Ap_4_A inhibits ADP-induced platelet activation [7], and a number of Ap_4_A analogs with modifications in the tetraphosphate chain have been synthesized and studied with the aim to improve on this effect and to increase the biological stability [8]–[10]. We recently reported that Ap_4_A and its P^1^- and/or P^4^-thio, and P^2^,P^3^-chloromethylene analogs inhibit platelet aggregation by targeting both P2Y_1_ and P2Y_12_ receptors [11], [12]. The most potent of these analogs for inhibition of platelet aggregation, diadenosine 5′,5″″-P^1^,P^4^-dithio-P^2^,P^3^-chloromethylenetetraphosphate, (compound **1**, Figure 1) [8], [12] has thio substitutions at the two terminal phosphate groups, which render the corresponding phosphorus atoms (P^1^ and P^4^) chiral. This, together with the pseudo-asymmetric carbon atom of chloromethylene group between P^2^ and P^3^ [13] gives rise to 4 stereoisomers for compound **1** [14] (See Discussion for details).

Phosphorothioate stereoisomers, in general, differ significantly in their substrate or ligand properties [15], [16], a fact which has been used extensively for various mechanistic studies [17]. Considering that platelet P2 receptors may exhibit stereoselectivity for the stereoisomers of **1** we preparatively separated its four diastereomers and studied their actions on ADP-induced human platelet aggregation, human platelet P2Y_1_ and P2Y_12_ receptors antagonism, and their stability and metabolism in human plasma. We found that the stereo configuration at P^1^ and P^4^ has significant effects on platelet aggregation, P2Y_12_ antagonism and plasma stability, and lesser effect on P2Y_1_ antagonism. Compounds with S_P_ configuration at both P^1^ and P^4^ have the highest platelet aggregation and P2Y_12_ inhibitory potency and highest stability in plasma.

## Materials and Methods

### Materials

Compound **1** was prepared as previously described [18]. Adenosine 5′-(P^1^-thio-P^2^,P^3^-chloromethylenetriphosphate), compound **2** was isolated as a minor byproduct during the synthesis of **1** and was characterized by ^1^H, and ^31^P NMR and liquid chromatography-mass spectrometry (LCMS). Adenosine 5′-thiomonophosphate (thioAMP), MRS2179, probenecid, adenosine 5′-(β,γ-methylenetriphosphate) (β,γ-CH_2_-ATP), ethylene glycol tetraacetic acid (EGTA) and apyrase (grade VII) were purchased from Sigma-Aldrich (St. Louis, MO). D-Phenylalanyl-L-prolyl-L-arginine chloromethyl ketone (PPACK) was purchased from Calbiochem (La Jolla, CA). FLUO-4 was from Invitrogen (Carlsbad, CA), ADP from Bio/Data (Horsham, PA), CD41-phycoerythrin (PE)-Cy5 from Beckman Coulter (Fullerton, CA). The VASP phosphorylation kit was purchased from BioCytex (Marseilles, France).

### Separation of the diastereomers of compound 1

Analytical separation of the diastereomers of compound **1** by reversed-phase high performance liquid chromatography (HPLC) has been described [14]. In the present work we used a modification of this method, utilizing a XBridge RP C18 3.5 µm, 4.6×150 mm column with a 4.6×10 mm guard column (Waters Inc., Waltham, Mass. USA), and a binary linear gradient from 100% of mobile phase A to 35% of mobile phase B in A for 35 min (A was 20 mM potassium phosphate buffer with pH 7, and B was 20% methanol in A) at 1 ml/min, 30°C column temperature, and UV detection at 260 nm.

Preparative separation was done on an XBridge C18 5 µm, 20×250 mm preparative column at 20 ml/min, all other conditions being the same as at the analytical runs. The fractions containing the separated diastereomers were pooled and concentrated under vacuum, and then desalted on the same column using volatile triethylammonium bicarbonate (TEAB) buffer, and a 15 min gradient from 0.2 M TEAB to 50% methanol in 0.2 M TEAB at 20 ml/min. The desalted fractions were evaporated under vacuum and the residual TEAB buffer was removed by repeated evaporation from methanol. Finally each diastereomer was converted to the sodium salts by passing through a column of Dowex W50X2 (5×20 mm) in the sodium form, followed by lyophilization. The isolated diastereomers were characterized by proton and phosphorous NMR and by mass spectrometry. Stock solutions (10 mM) in water were prepared using molar absorptivity of 20.2 mM^−1^cm^−1^ at 260 nm [8], and were stored frozen at −80°C.

### Ethics Statement

This study was approved by the University of Massachusetts Medical School Institutional Review Board (IRB). Written IRB-approved informed consent was obtained prior to blood collection.

### Blood collection and sample preparation

Human blood samples were taken from healthy volunteer donors free from aspirin or other non-steroidal anti-inflammatory drugs for more than 7 days. Blood was drawn from antecubital veins into tubes containing 3.2% sodium citrate. For platelet aggregation assays the blood was centrifuged at 110× g for 12 minutes, and platelet-rich plasma (PRP) was immediately removed. Centrifugation at 1650× g for 10 minutes was to obtain platelet-poor plasma (PPP).

### ADP-induced platelet aggregation

The 96-well microplate method for the detection of ADP-induced platelet aggregation and the concentration dependence of its inhibition by the tested compounds was used as previously described [11], [12], thereby avoiding the problem of platelet aging [19], [20]. In brief, PRP at 37°C was added to a pre-warmed 96-well microplate containing ADP (3 µM final concentration) and test compounds (various concentrations) or vehicle (10 mM Hepes, 0.15 M NaCl, pH 7.4). Light transmission at 580 nm was recorded immediately and at 11 second intervals for 6 min at 37°C with intermittent programmed shaking of the plate in a Molecular Devices microplate reader. Within each experiment all samples were run in duplicate and each experiment was repeated 3–5 times with PRP from different donors.

### P2Y_12_-mediated vasodilator-stimulated phosphoprotein (VASP) phosphorylation assay

VASP phosphorylation was measured by flow cytometry using a BioCytex kit, essentially according to the manufacturer's recommendations, except that a small volume of the test compound solution or vehicle (HEPES-saline) was added to each assay tube as previously described [11], [12]. Analysis was performed in a FACSCalibur (Becton Dickinson) flow cytometer.

### P2Y_1_-mediated cytosolic Ca^2+^ increase assay

ADP-dependent, P2Y_1_-mediated increase in platelet cytosolic Ca^2+^ was measured by detecting changes in FLUO-4 fluorescence as previously described [11], [12]. In brief, citrated whole blood was added to a loading solution consisting of 5 µM FLUO-4, CD41-PE-Cy5 and 1 mM probenecid, and the mixture was incubated for 30 minutes at room temperature. Samples were diluted 36-fold in 10 mM HEPES, 0.15 M NaCl, pH 7.4 and analyzed in a FACSCalibur flow cytometer. After obtaining a 30 second baseline recording, the acquisition was paused, and 60 µL of ADP (3 µM final concentration) and test compound solutions at various concentrations or ADP plus vehicle (HEPES-saline) were quickly added, the sample mixed, and the acquisition resumed (total pause time less than 10 seconds). FLUO-4 fluorescence before and after addition of ADP (3 µM final concentration) and test compound solutions was monitored. The mean FLUO-4 fluorescence of the baseline 30-second interval and of 10-second post-stimulant intervals was calculated. The cytosolic Ca^2+^ increase was calculated as the ratio of the maximal post-stimulant FLUO-4 fluorescence to the baseline FLUO-4 fluorescence. The percent inhibition of ADP-induced Ca^2+^ increase due to the addition of the test compounds was calculated relative to 3 µM ADP plus vehicle (HEPES-saline).

### Stability and metabolism in plasma

Frozen, pooled, heparin-anticoagulated human plasma (BioReclamation, Westbury, NY, catalog # HMPLNAHP) was thawed upon arrival, aliquoted (2.5 ml) in sterile polypropylene vials, re-frozen in dry ice, and stored at −20°C. At the time of testing the plasma aliquots were thawed and incubated at 37°C for 10 minutes. Test compound, 25 µL of 10 mM solution in water (100 µM final concentration) was added, the sample was mixed briefly, and incubated at 37°C. Aliquots (100 µL) were taken at the indicated times, mixed with 35% HClO_4_ (8 µL), and centrifuged (10 min, 13000× g). Forty µL supernatant were removed, mixed with 160 µL of 50 mM K_2_CO_3_, and centrifuged (15 min, 13000× g). Supernatant, 150 µL was removed for HPLC analysis. The chemical stability of the test compounds under the incubation and work up conditions was controlled by incubation with heat-inactivated (10 minutes at 95°C) plasma. Plasma incubation without test compound addition was used for method specificity control. The external standard calibration was done using plasma samples, spiked with test compounds, and processed without incubation. The extraction efficiency was evaluated by analysis of the calibration samples relative to the analysis of standard samples in mobile phase, and was above 90%. The main metabolites, the diastereomers of compound **2** (Figure 1) and thioAMP, were identified by LCMS analysis of incubation mixtures, and confirmed by analysis of heat-inactivated plasma samples spiked with thioAMP and synthetic compound **2**.

### Statistical analysis

The results were analyzed using GraphPAD Prism software, version 4.00 for Windows (GraphPad Software, San Diego, CA). All data are expressed as mean followed by 95% confidence interval (95% CI). Student's t-test was used to determine statistical significance when two groups of data were compared. One-way ANOVA and Bonferroni's multiple comparison tests were used when three or more groups of data were compared. The rate constants for degradation in plasma and the associated half-lives were estimated by a non-linear fitting of first order elimination model to the chromatographic peak areas.

## Results

### Separation of diastereomers

**Figure 2:**
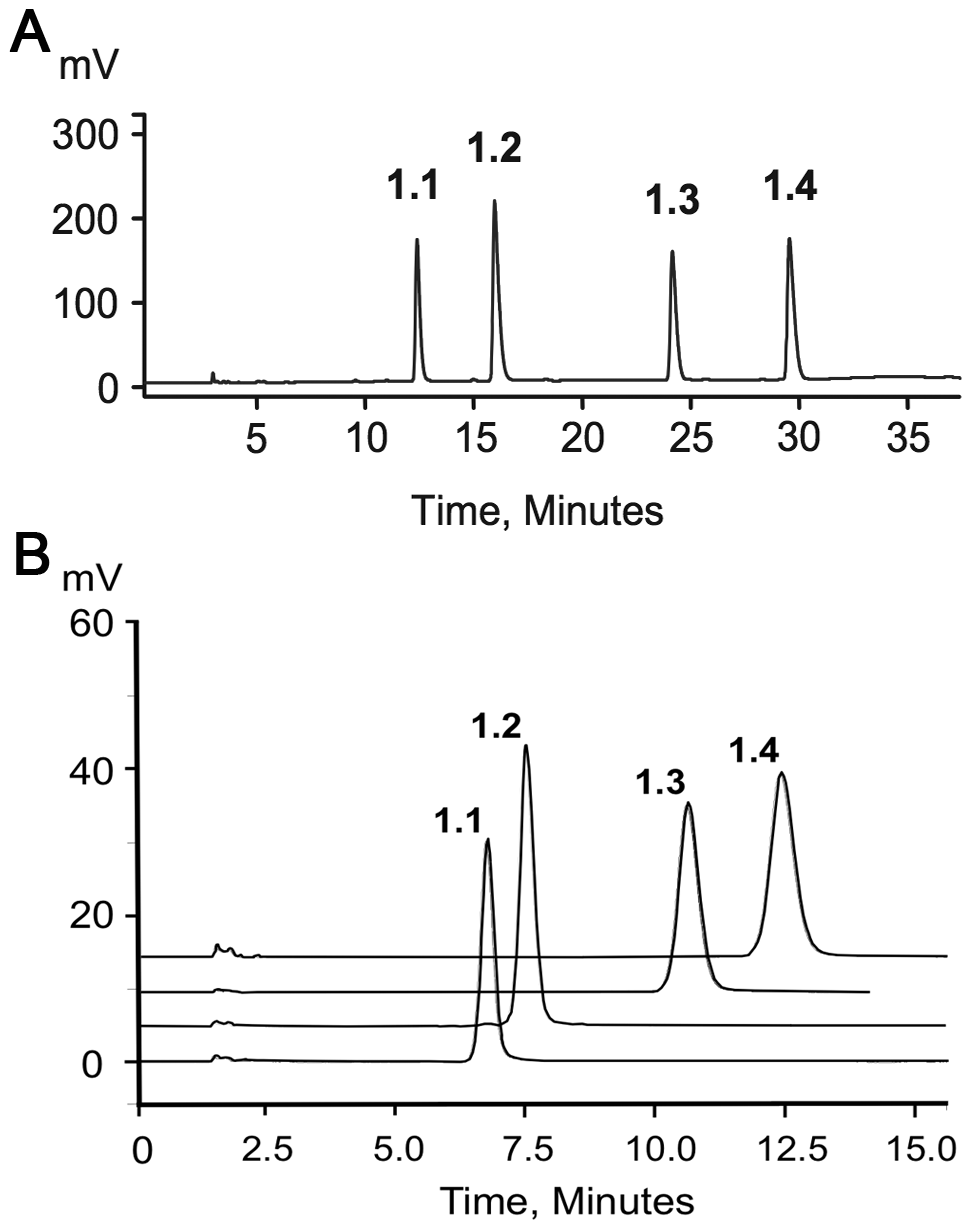
Reversed-phase HPLC separation of the diastereomers of compound 1. Panel A: chromatogram of the racemic mix of the diastereomers of compound **1** analyzed by reversed-phase HPLC using a XBridge C18 column, 3.5 µm, 4.6×150 mm (Waters Inc., Waltham, Mass. USA), linear gradient from 1 to 7% methanol in 20 mM potassium phosphate buffer, pH 7, 1 ml/min, detection by UV at 260 nm; Panel B: chromatograms of isolated individual diastereomers (**1.1**–**1.4**) of compound **1** analyzed using the same column and flow rate, but with isocratic elution with 7% methanol in 20 mM potassium phosphate buffer, pH 7.

**Figure 3:**
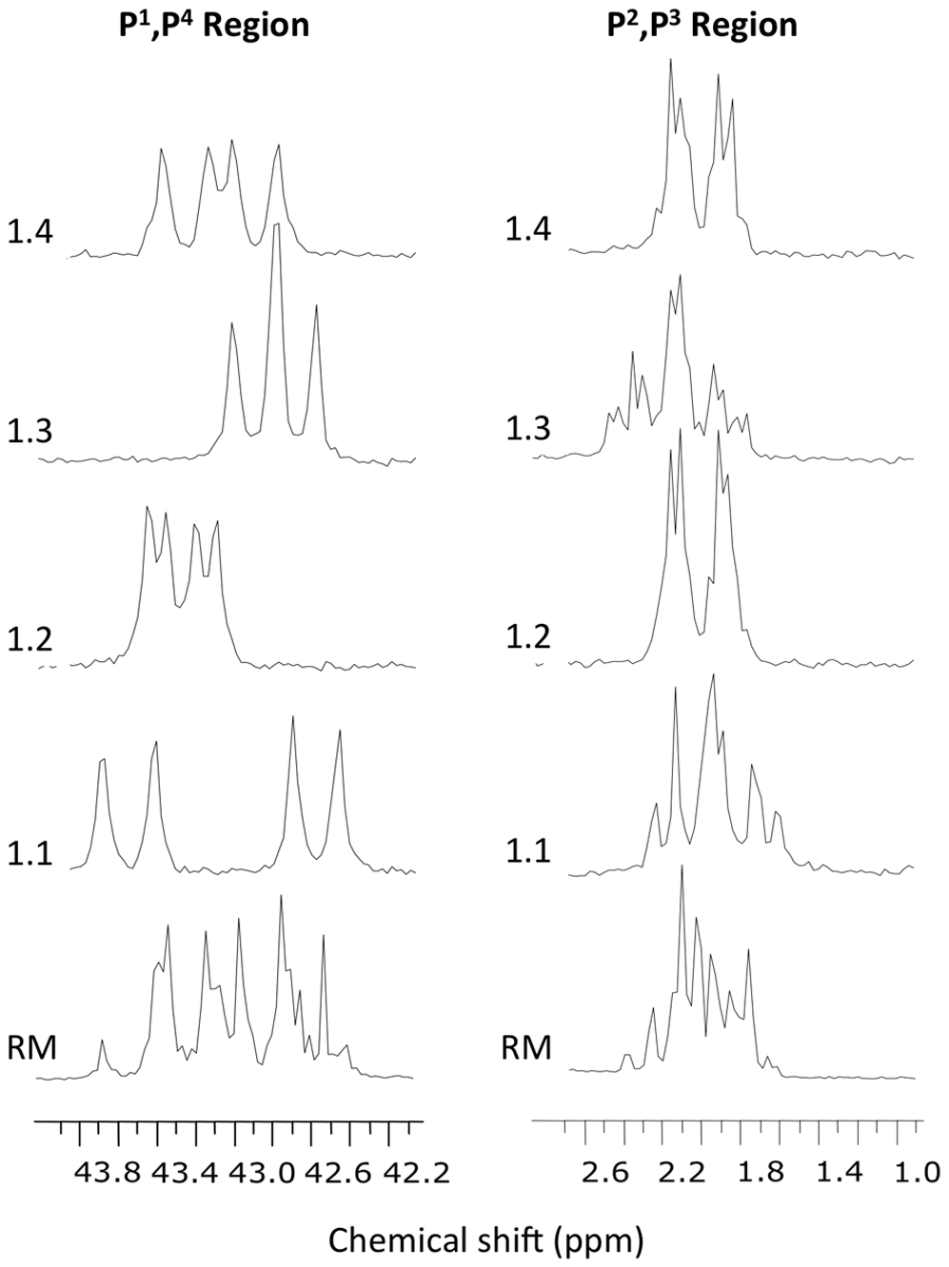
^31^P NMR Spectra (^1^H decoupled) of the diastereomers of compound 1 and their racemic mixture. In the left panel are the P^1^,P^4^ regions, and in the right panel are the P^2^,P^3^ regions of the spectra. The traces are, from bottom to top: racemic mixture (RM), diastereomer **1.1**, **1.2**, **1.3**, and **1.4**.

Excellent separation of the diastereomers of compound **1** was achieved with 20 mM potassium phosphate buffer at very low (1–7%) methanol content on a C18 column (Figure 2A). The optimal pH of the buffer was from 7 to 8. The peak shape quickly deteriorated at pH values below 6. This method scaled up well on preparative XBridge C18 5 µm column (25×250 mm). After desalting and conversion to their sodium salts, 1.7, 3.3, 3.8, and 2.4 mg of diastereomers **1.1**, **1.2**, **1.3**, and **1.4**, respectively, were prepared. The purity of each diastereomer was above 95% by analytical HPLC (Figure 2B). By mass spectroscopy, the isolated diastereomers were all identical and indistinguishable from the racemic mixture (data not shown). Characterization by phosphorous NMR showed distinct resonance profiles in the P^1^,P^4^ and P^2^,P^3^ regions for each of the isolated diastereomers (Figure 3) consistent with their high purity. The most significant differences in the proton NMR spectra were in the resonances of the proton of the monochloromethylene group (see structure in Figure 1), which, due to the two bond coupling to P^2^ and P^3^, were observed as characteristic triplets (^2^J_P-H_ = 17.3–17.7 Hz) at 4.675 (**1.1**), 4.454 (**1.2**), 4.825 (**1.3**), and 4.525 (**1.4**) ppm.

The absolute configurations of the phosphorothioate groups of the four diastereomers of compound **1** (Figs. 1 and 2) have already been assigned [14] by using snake venom phosphodiesterase digestion (See Discussion section for details).

### Inhibition of ADP-induced platelet aggregation

**Figure 4:**
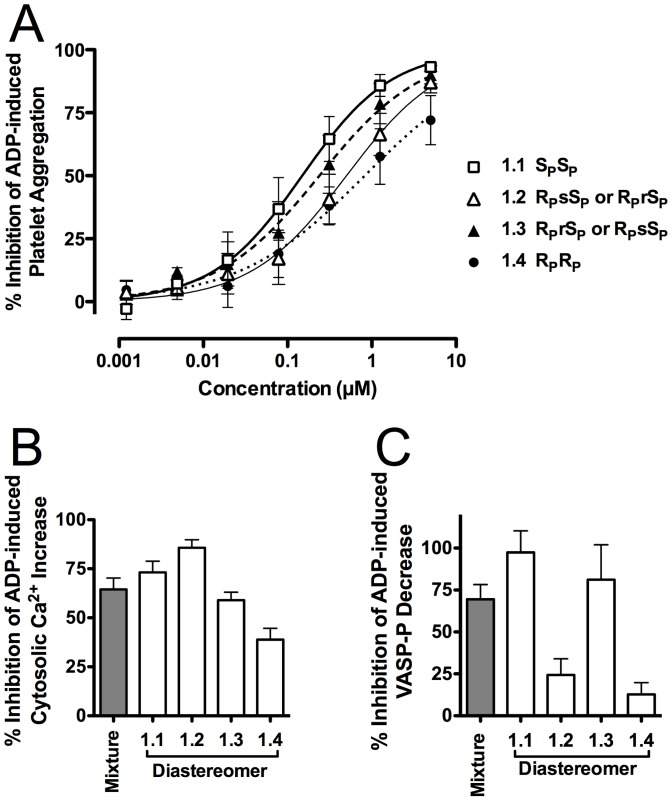
Effects of compound 1 diastereomers on platelet aggregation and platelet ADP receptor signaling. Inhibition by the diastereomers of compound **1** of: A, 3 µM ADP-stimulated platelet aggregation; B, P2Y_12_ mediated ADP-induced decrease of VASP phosphorylation; and C, P2Y_1_-mediated ADP-induced intraplatelet Ca^2+^ level increase. VASP, vasodilator-stimulated phosphoprotein. For other abbreviation, see Figure 1.

Figure 4A shows the dose response curves, determined in parallel, of the four diastereomers of compound **1** for inhibition of 3 µM ADP-induced platelet aggregation. The IC_50_s (listed in Table 1) for diastereomers **1.1** and **1.3** were not significantly different from each other. The IC_50_s for diastereomers **1.2** and **1.4** also did not differ significantly from each other, but were both significantly higher (p<0.0001, F-test) than those for **1.1** and **1.3**. Therefore, the antiaggregatory potency order was **1.1**≈**1.3**>**1.2**≈**1.4**.

**Table 1:** Platelet-related properties of the four diastereomers of compound **1**.

Diastereomer number and configuration	Platelet Aggregation (IC_50_, µM, 95% CI)	P2Y_12_ VASP^a^ Phosphorylation (% inhibition at 10 µM, 95% CI, n = 3)	P2Y_1_ Calcium Flux (% inhibition at 10 µM, 95% CI, n = 6)
**1.1**, S_P_S_P_	0.15 (0.09 to 0.24)	97.4 (42.2 to 152.7)	73.2 (58.5 to 87.8)
**1.2**, S_P_sR_P_≡S_P_rR_P_	0.50 (0.32 to 0.79)^b^^,^ e	24.3 (−17.2 to 65.9)^c^	85.7 (75.1 to 96.3)
**1.3**, S_P_rR_P_≡S_P_sR_P_	0.24 (0.14 to 0.40)	81.2 (−8.4 to 170.8)	59 (48.5 to 69.5)
**1.4**, R_P_R_P_	0.82 (0.45 to 1.50)^e^	12.8 (−17.3 to 42.9)^d^	38.9 (24.1 to 53.6)^e^

a VASP, vasodilator-stimulated phosphoprotein.

b All comparisons *vs.* diastereomer **1.1**.

c p<0.05.

d p<0.01.

e p<0.001.

### Antagonism of platelet P2Y_12_-mediated, ADP-induced decrease in VASP phosphorylation

The relative potency of the four diastereomers to inhibit platelet P2Y_12_ receptors was tested by their ability, at a single concentration of 10 µM, to inhibit the ADP-induced decrease of VASP phosphorylation, an index of platelet P2Y_12_ receptor activation [11], [12]. The percent inhibitions are shown in Figure 4B and Table 1. Diastereomer **1.1** was significantly more potent than **1.2** (p<0.05) and **1.4** (p<0.01, Bonferroni's multiple comparison test), and did not differ significantly from **1.3**. The IC_50_s for diastereomers **1.2** and **1.4** also did not differ significantly from each other. The order of potency was **1.1**≈**1.3**>**1.2**≈**1.4**.

### Antagonism of platelet P2Y_1_-mediated, ADP-induced increase in cytosolic Ca^2+^

The relative potency of the individual diastereomers of compound **1** to inhibit platelet P2Y_1_ receptors was evaluated at the single concentration of 10 µM by their ability to reduce 3 µM ADP-induced intracellular Ca^2+^ increase due to P2Y_1_-mediated Ca^2+^ release from intraplatelet stores. The percent inhibitions are shown in Figure 4C and Table 1. Compared with diastereomer **1.1**, diastereomer **1.4** was significantly less potent for inhibition of platelet P2Y_1_ function (p<0.05, n = 6, Bonferroni's multiple comparison test) while **1.2** and **1.3** did not differ significantly from **1.1**. The order of potency was **1.2**>**1.1**>**1.3**>**1.4**.

### Metabolism in human plasma

**Figure 5:**
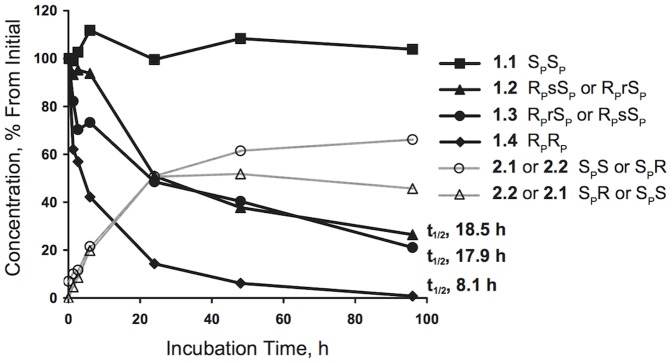
Stability and metabolism of the four diastereomers of compound 1 in human plasma at 37 °C. t_1/2_, half-life of first order elimination. For other abbreviations, see Figure 1.

Although compound **1** is very stable in human plasma, by using very long incubation times we were able to observe clear differences in the rate of degradation and the type of degradation products of its diastereomers (Figure 5). Diastereomer **1.4** degraded to thioAMP with a half-life (t_1/2_) of 8.1 hours. Diastereomers **1.2** and **1.3** degraded with almost equal rates (t_1/2_ 17.9 and 18.5 hours, respectively) giving equal amounts of thioAMP and two different stereoisomers of the triphosphate **2**, which were resistant to further hydrolysis. Diastereomer **1.1** was completely stable in human plasma, with no loss after 96 hours incubation at 37°C. In fact we did not observe measurable degradation of this diastereomer even after incubation for 6 days at 37°C (data not shown). Also, control incubation of the diastereomeric mixture of **1** at 37°C with heat denatured human plasma for 6 days showed no decomposition, demonstrating the remarkable chemical stability of compound **1**. The overall order of stability of the four diastereomers of compound **1** in human plasma was **1.1**>**1.2**≈**1.3**>**1.4**.

## Discussion

### Stereoisomers of compound 1 and their absolute configuration assignment

The absolute configuration of a phosphorous atoms containing 4 different substituents is designated, according to Ingold-Kahn-Prelog notation, as R_P_ or S_P_ [21]. The subscript “P” indicates that this absolute configuration applies to an asymmetric phosphorous, and not to a carbon atom. The stereochemical character of the chloromethylene group in compound **1** depends on the absolute configuration at P^1^ and P^4^. When P^1^ and P^4^ have the same configuration (R_P_R_P_, S_P_S_P_) the carbon atom of the chloromethylene group has two identical substituents, and is not asymmetric. However, when P^1^ and P^4^ are of different configurations (R_P_S_P_≡S_P_R_P_), this carbon atom becomes pseudo-asymmetric [13], and can exist in two absolute configurations designated with r and s. The prefix “pseudo” indicates that this carbon contains two substituents that differ only in their stereo-configuration, and lower case r and s, instead of upper case R and S are used for the same reason. Therefore compound **1** can have 4 different stereoisomers with configurations S_P_S_P_, S_P_sR_P_≡R_P_sS_P_, S_P_rR_P_≡R_P_rS_P_, and R_P_R_P_ (S_P_sR_P_ is identical to R_P_sS_P_, and S_P_rR_P_ is identical to R_P_rS_P_ because of the symmetry of the molecule relative the chloromethylene group, Figure 1). All those stereoisomers are diastereomeric to each other because of the chiral character of the ribose moiety. Blackburn et al. [14], [22] used digestion with snake venom phosphodiesterase (SVP), and the well established resistance of phosphorothioates in S_P_ configuration to hydrolysis by this enzyme [23], [24] to assign the absolute configurations of the four diastereomers of compound **1** and its closely related monofluoromethylene analog in the order of their elution (Figure 2) as: **1.1**, S_P_S_P_; **1.2**, S_P_sR_P_ or S_P_rR_P_; **1.3**, S_P_rR_P_ or S_P_sR_P_, and **1.4**, R_P_R_P_. These assignments are also in agreement with the well known observation that S_P_ phosphorothioates elute earlier the R_P_ isomers [23], [24]. The SVP digestion method does not allow for the determination of the absolute configuration of the pseudo-asymmetric carbon atom between P^2^ and P^3^.

### Inhibition of ADP-induced platelet aggregation and platelet P2Y_1_ and P2Y_12_ receptors

Well-defined differences in inhibition of ADP-induced platelet aggregation and platelet P2Y_1_ and P2Y_12_ antagonist properties of the four diastereomers of compound **1** were observed (Figure 4 and Table 1). The diastereomers of compound **1** could be separated into two groups based on their potency for inhibition of platelet aggregation: **1.1, 1.3** and **1.2, 1.4**. The differences between the IC_50_s within each pair are not statistically significant, but between the pairs the differences are statistically significant, **1.1** and **1.3** being significantly more active than **1.2** and **1.4**. This same order of potency was observed for the antagonist activity of the diastereomers toward P2Y_12_ (Figure 4B), where **1.1** and **1.3** are significantly more active than **1.2** and **1.4**. In contrast, **1.2** inhibited P2Y_1_ with potency similar to that of **1.1** and **1.3** and greater than that of **1.4** (Figure 4C), yet it had reduced potency for inhibition of platelet aggregation (Figure 4A). Compound **1.4**, showed significantly reduced antagonist activity towards both P2Y_1_ and P2Y_12_, and had the highest IC_50_ for inhibition of platelet aggregation.

Taken together, these results suggest that for optimal platelet aggregation and platelet P2Y_1_ and P2Y_12_ receptor inhibition *at least one* of the P^1^ and P^4^ phosphorothioates needs to be in the S_P_ configuration. Structurally, **1.2** and **1.3** differ only in the configuration of the carbon atom of the chloromethylene group, but differ significantly in their ability to antagonize P2Y_12_. Because the absolute configuration of this carbon atom is assigned arbitrarily to r or s, we cannot presently ascertain the absolute configuration of the chloromethylene group that favors P2Y_12_ antagonism.

In the case of P2Y_1_, **1.4** (R_P_R_P_ configuration) is the least active, yet the difference between all diastereomers, and especially between **1.2** and **1.3**, is less pronounced if compared with the differences in their activity observed for P2Y_12_ receptors.

Even though chiral phosphorothioate nucleotides have been extensively used as probes for structural and mechanistic studies of enzymes and receptors [15]–[17], and the question of molecular recognition of P2Y_12_ receptors has been addressed in numerous works (e.g., see [25], [26] and references cited therein), to the best of our knowledge the question of P2Y_12_ stereoselectivity has not been addressed. Major et al. [27] studied P2Y_1_ receptors stereoselectivity using the diastereomers of α-thioATP, α-thio-2-thiomethylATP, and α-thio-2-chloroATPs as agonists of human P2Y_1_ receptors in HEK cells. The diastereomers with S_P_ configuration displayed 8–20 fold higher agonist activity than the R_P_ diastereomers. Assuming a similar mode of binding and identical binding sites for ATP and Ap_4_A analogs, those results would corroborate our conclusions for the preference of P2Y_1_ receptors for the S_P_ configuration at P^1^.

In the present study we observed better correlation between P2Y_12_ inhibition and platelet aggregation inhibition than between P2Y_1_ inhibition and platelet aggregation inhibition for the four diastereomers of compound **1**. Yet, in our opinion, this observation cannot be used to discount a possible contribution of P2Y_1_ inhibition to the overall platelet aggregation inhibition by these compounds, especially taking into account the relatively small number of binding sites of the P2Y_1_ receptors on the platelet surface [28], and further studies will be necessary to shed light on the relative contribution of the inhibition of each of the receptors.

### Stereoselectivity in the plasma metabolism of the diastereomers of compound 1

Because of the important physiological functions of dinucleoside polyphosphates their intra- and extracellular levels are tightly regulated [29]. Extracellular dinucleoside polyphosphates are hydrolyzed by ecto-nucleotide pyrophosphatase/phosphodiesterases 1–3 (NPP1, PC-1; NPP2, autotoxin; NPP3, Gp130) [30]. Despite the important physiological role of NPP1-3, little is known about their stereospecificity. Koziolkiewicz et al. [31] determined that 3′-exonuclease present in human plasma degrades phosphorothioate oligonucleotides with the R_P_ configuration, whereas the S_P_ configuration is resistant to hydrolysis. More recently, Wojcik et al. [32] showed that the enzyme responsible for this action is NPP1 (PC-1). The present results show that the degree of the stabilization due to the thio modification depends significantly on its stereo configuration, and that the phosphorothioate group in the S_P_ configuration is resistant to the hydrolytic action of those enzymes. Thus compound **1.4**, having both P^1^ and P^4^ in the R_P_ configuration was the least stable diastereomer, presumably being degraded first to one mole of thioAMP and one mole of compound **2** in the R_P_ configuration (actually, equal amount of diastereomers **2.3** and **2.4**, see Figure 1). Both **2.3** and **2.4** have a phosphorothioate group in the R_p_ configuration, and are further quickly degraded to thioAMP and chloromethylene-*bis*-phosphonate. Diastereomers **1.2** and **1.3**, which both have one phosphorothioate group in the R_P_, and one in the S_P_ configuration, were hydrolyzed about 2 times slower than **1.4** to equimolar amount of thioAMP and two stereo isomers of compound **2**, which were stable to further hydrolysis. This suggests that both **1.2** and **1.3** are hydrolyzed at the thiophosphate group in the R_P_ configuration, giving the S_P_S and S_P_R isomers of **2** (**2.1** and **2.2**, Figure 1), which both have their phosphorothioate group in the S_p_ configuration, and resist further hydrolysis. Finally, **1.1**, having both P^1^ and P^4^ phosphorothioate groups in the S_P_ configuration did not show signs of hydrolysis even after very long (up to 6 days) incubations in human plasma.

The physiologically important function of the ecto-nucleotide pyrophosphatase/phosphodiesterase enzymes in regulating and spatially modulating various purinergic signaling pathways, and their value as therapeutic targets have been increasingly realized [33], [34], which makes the development of inhibitors or high affinity probes of the NPP class an important research direction. Compound **1.1** could be utilized as a convenient starting point for that purpose. Our observation that **1.1** was completely resistant to plasma degradation even after very long incubations opens the possibility of crystallizing its complex with NPPs, thus providing additional information for this important class of enzymes.

P2Y_12_ is among the most important targets of antiplatelet drugs in current use and under development [35]. The potential of P2Y_1_ as a target for new antiplatelet drugs, or agents regulating the vascular inflammation process has been recognized [36]. The information in this study can be used to aid in elucidating the structure and the mode of action of these targets, and in the design of new inhibitors of scientific and/or therapeutic interest – specifically inhibitors of both P2Y_1_ and P2Y_12_.
